# Bile cast nephropathy in a patient with cholangiocarcinoma – a case report

**DOI:** 10.1002/ccr3.1465

**Published:** 2018-03-05

**Authors:** Kittrawee Kritmetapak, Thanatarn Sathidatekoonchorn, Weerapat Papanrueng

**Affiliations:** ^1^ Division of Nephrology Faculty of Medicine Khon Kaen University Khon Kaen Thailand; ^2^ Department of Medicine Faculty of Medicine Khon Kaen University Khon Kaen Thailand

**Keywords:** Bile cast nephropathy, renal failure, proximal tubulopathy, hyperbilirubinemia

## Abstract

Bile cast nephropathy is characterized by the presence of bile casts associated with renal failure and/or proximal tubulopathy in cases of severe hyperbilirubinemia. The clinician should carefully examine the urine samples for characteristic bile‐stained granular casts in suspected case.

## Introduction

Cholangiocarcinoma is one of the most common cancers in Thailand, especially in the northeastern region. Patients typically present with progressive jaundice and may also develop renal failure. The etiologies of acute kidney injury in a jaundiced patient commonly include hypovolemia, infection, hepatorenal syndrome, and acute tubular necrosis. Severely high serum bilirubin levels can also compromise renal function and lead to renal failure. Heretofore, this was regarded as cholemic nephrosis (bile nephrosis) but nowadays is more commonly referred to as “bile cast nephropathy.” The contribution of this condition to our perception of the various mechanisms of renal impairment in jaundiced patients has usually been overlooked. Herein, the authors report a case of newly diagnosed cholangiocarcinoma and oliguric renal failure due to bile cast nephropathy and give a brief review of its pathophysiology.

## Case Report

A 63‐year‐old Thai man, with a long‐standing history of alcohol and tobacco use, was hospitalized with a two‐month history of progressive jaundice, generalized pruritus, and significant involuntary weight loss. He reported no fever, abdominal pain, or vomiting. For the 4 weeks prior to his admission, he also suffered from progressive fatigue, poor oral intake, pale stools, and decreased urine output. His past medical history was significant for well‐controlled type 2 diabetes mellitus, hypertension, and stage 3A chronic kidney disease (baseline serum creatinine of 1.5 mg/dL, estimated glomerular filtration rate of 48.8 mL/min/1.73 m^2^). His family history was negative for any kidney disease or malignancy. He had no history of herbal medicine use, intravenous drug abuse, tattoos, or blood transfusions. On physical examination, he was alert and oriented with severe jaundice, a distended abdomen with an enlarged liver, and a 1+ bilateral pitting edema of his lower extremities. He had no stigmata of chronic liver disease. The rest of the physical examination was unremarkable.

**Figure 1:**
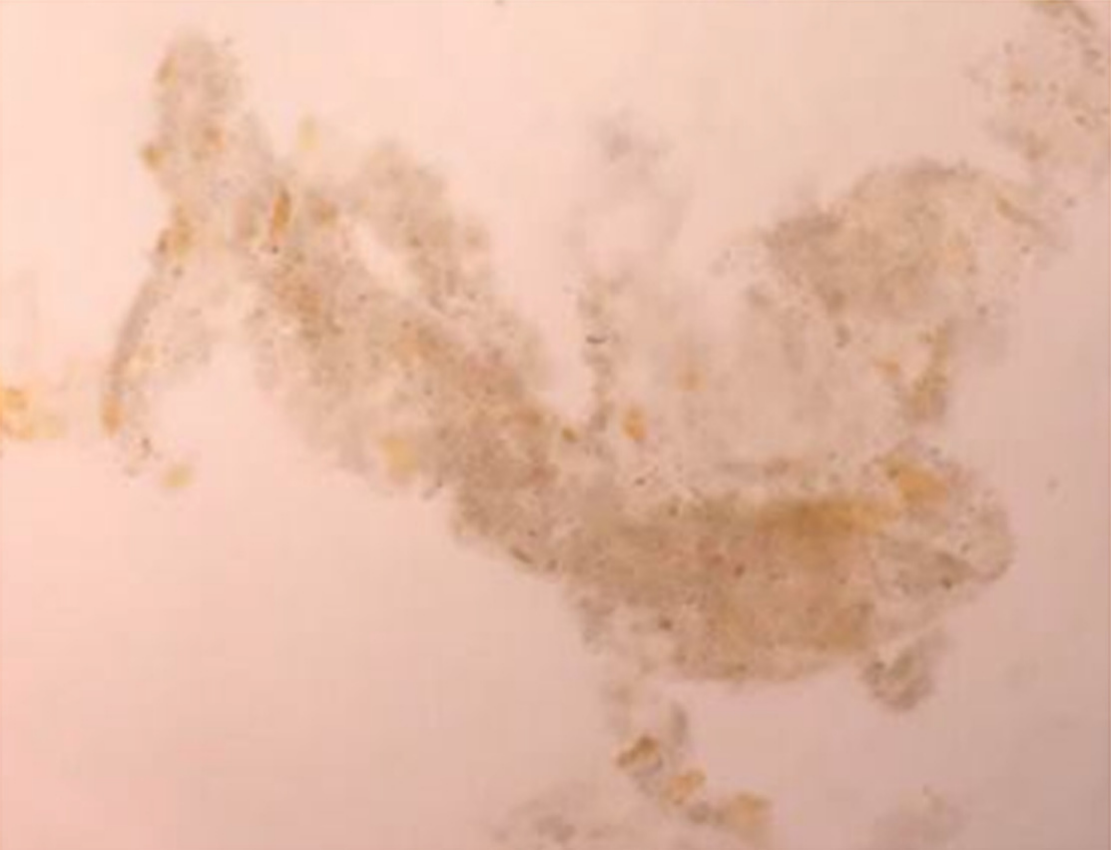
Urine microscopy showing the presence of muddy brown granular casts with bile staining (400x magnification).

**Figure 2:**
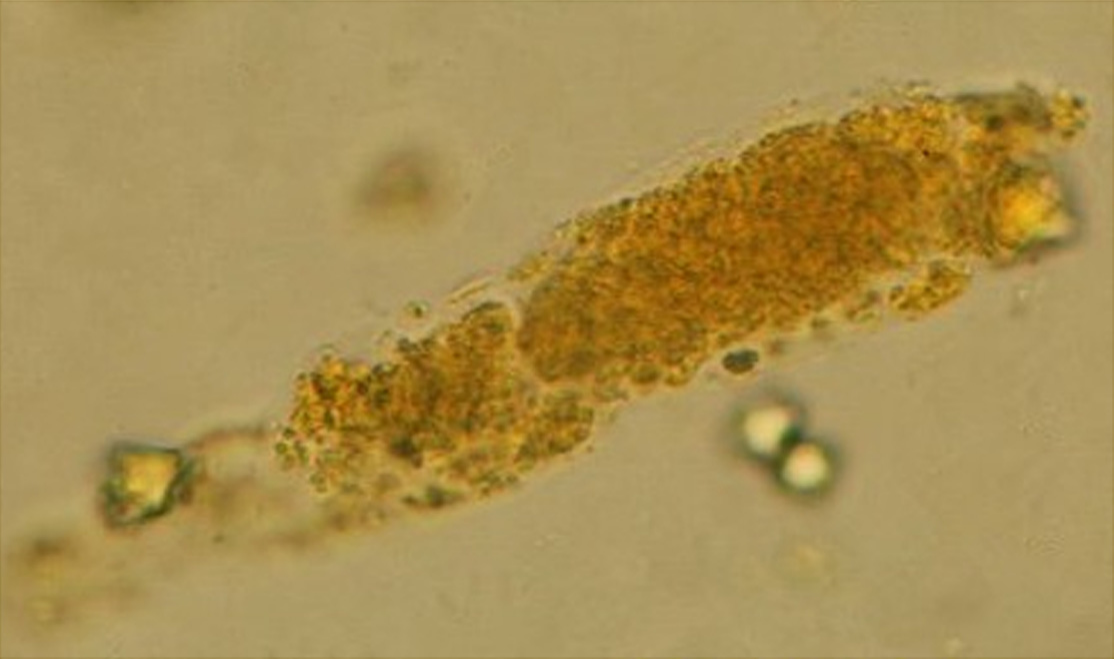
Urine microscopy showing the presence of bile cast (400x magnification).

**Figure 3:**
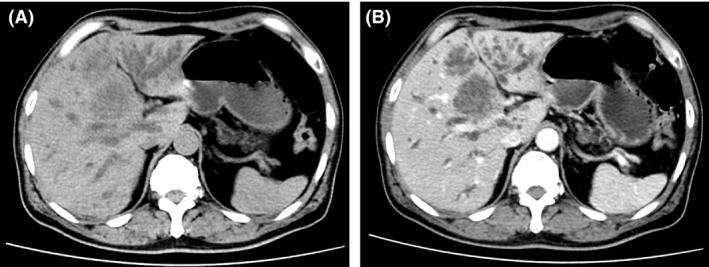
Mass‐forming cholangiocarcinoma. The axial unenhanced abdominal computed tomography image shows two ill‐defined low‐density masses at right lobe of the liver with moderately dilated intrahepatic ducts (A), and these masses show minimal peripheral enhancement in the arterial phase (B).

Laboratory data were as follows: hemoglobin = 9.6 g/dL, mean corpuscular volume (MCV) = 85 fL, white blood cells = 11,880/mm^3^ (neutrophil 81%, lymphocyte 4.9%, monocyte 12%), platelets = 384,000/mm^3^, international normalized ratio (INR) = 2.2, blood urea nitrogen = 73.8 mg/dL, serum creatinine = 7.78 mg/dL, sodium = 133 mEq/L, potassium = 3.8 mEq/L, bicarbonate = 16.8 mEq/L, chloride = 92 mEq/L, calcium = 8.7 mg/dL, phosphorus = 4.8 mg/dL, magnesium = 2.8 mg/dL, and glycated hemoglobin = 7.1%. Liver function tests revealed the following: total cholesterol = 177 mg/dL, total protein = 5.4 g/dL, albumin = 2.8 g/dL, globulin = 2.6 g/dL, total bilirubin = 36.1 mg/dL, direct bilirubin = 35 mg/dL, alanine transaminase (ALT) = 53 U/L, aspartate transaminase (AST) = 109 U/L, and alkaline phosphatase (ALP) = 408 U/L. Serum tests for human immunodeficiency virus, hepatitis B, and hepatitis C were negative. Serum carbohydrate antigen 19‐9 (CA 19‐9) levels were 850 U/mL (0–37). Urine appeared greenish with a specific gravity of 1.010, trace proteinuria, 5–10 red blood cells (RBCs)/high‐power field, 1–2 white blood cells/high‐power field along with numerous muddy brown granular casts (Fig. 1), and bile casts (Fig. 2). No RBC casts were found. The fractional excretion of sodium (FE_Na_) was 2.7%, and FE_urea_ was 44.8%. Abdominal computed tomography showed 4.3 × 4.2 cm and 3.1 × 3.5 cm inhomogeneous enhancing masses at liver segment 4A with moderately dilated intrahepatic ducts (Fig. 3). Based on the symptoms of biliary obstruction, typical imaging findings, and elevated serum CA 19‐9, the most likely diagnosis was cholangiocarcinoma (Bismuth–Corlette type IIIb). He was initially given intravenous fluids to combat his oliguric renal failure, but there was no improvement in serum creatinine and urine output. Over the next 5 days, hemodialysis was initiated due to diuretic‐resistant oliguric acute renal failure. Percutaneous transhepatic biliary drainage (PTBD) was also performed to relieve his obstructive jaundice. However, he still experienced clinical deterioration. After approximately 2 weeks of hemodialysis, his total serum bilirubin continued to be in the 30 mg/dL range and he remained oliguric. On the 27th day of admission, the patient suffered from massive biliary hemorrhage requiring a blood transfusion. An emergency esophagogastroduodenoscopy (EGD) was performed and revealed active hemorrhage from the ampulla of Vater, a finding suggestive of tumor bleeding. After thorough discussion and consultation between the patient's family and the palliative care team, the healthcare providers proceed to initiate palliative treatment.

## Discussion

We report on the case of a patient with newly diagnosed cholangiocarcinoma who had high total serum bilirubin levels, developed oliguric renal failure due to presumed bile cast nephropathy, and finally expired from intractable upper gastrointestinal hemorrhage.

Cholemic nephrosis, or bile nephrosis, has been described since 1899 1, 2. The term “cholemic nephrosis” heralds the presence of renal injury secondary to the components of bile. In 2006, Betjes et al. 3 proposed the term “jaundice‐related nephropathy” for what had formerly been called “cholemic nephrosis” – alterations varying from proximal tubulopathy to renal failure owing to the accumulation of bile and bile salts. Subsequently, van Slambrouck et al. 4 proposed the term “bile cast nephropathy” be used to specifically designate the presence of bile casts promoting renal tubular injury and mechanical obstruction. Bile casts are quite similar to “myeloma” casts or myoglobin casts because both of these also have direct deleterious effects on renal tubular epithelial cells and an obstructive capability 5, 6, 7. Noteworthy, the bile casts are typically not present in cases of hemolytic jaundice as the type of bilirubin in these cases is unconjugated and generally considered nontoxic. Moreover, the pathologic casts recognized in kidney biopsies of patients with hemolysis‐associated acute kidney injury are mainly of hemoglobin within proximal tubular epithelial cells and intratubular hemoglobin casts in distal nephron 8.

Bile cast nephropathy is usually found in patients with advanced liver disease concomitant with cholestasis. However, there are few reports available on the relationship between cholangiocarcinoma and bile cast nephropathy. It is evident that renal failure is common in cases of cholangiocarcinoma, and it is previously believed due to acute tubular necrosis. Nonetheless, bile cast nephropathy in retrospect may have been a significant contributing factor to the acute kidney injury in this clinical setting. A large retrospective cohort study by Mairiang et al. reported that the duration of jaundice before the onset of renal failure was prolonged, averaging 40 days which is compatible with the case presented here. There are multiple causes of renal failure in cholangiocarcinoma, and hemodynamic derangement contributes remarkably to the development of renal impairment. The contributing factors consist of increased sensitivity to catecholamines, renin‐angiotensin‐aldosterone stimulation, prostaglandin‐induced hypotension, cardiac dysfunction, and endotoxemia‐induced cytokine release 9.

Bile cast nephropathy mostly constitutes a wide spectrum of renal disease, from modest reversible alterations in those without underlying renal impairment and hyperbilirubinemia of short duration, to permanent renal failure in those with underlying renal dysfunction with severe, long‐standing hyperbilirubinemia 10. The risk of acute kidney injury significantly increases when total serum bilirubin level rises above 20 mg/dL particularly in the existence of hypoalbuminemia and acidosis 10, 11, 12, 13. This predisposes to diminished binding of bilirubin and bile acids to albumin, by which allowing them to be freely filtered by the glomerulus with later augmented renal tubular exposure. It has been presumed that there is a limitation to the amount of bilirubin that can be transported in the proximal tubular cells, after which they become saturated, contributing to bile cast formation and intratubular obstruction 14. The modest water solubility of bile acids may also facilitate cast formation within the acidic environment of the distal nephron 15. Proximal tubular dysfunction may appear before structural alterations showing as glycosuria, amino aciduria, phosphaturia, bicarbonaturia, uricosuria, and low molecular weight proteinuria 16. The mechanisms accountable for renal tubular dysfunction encompass uncoupling of mitochondrial phosphorylation by bilirubin and oxidative injury of tubular cell membranes together with suppression of apical Na^+^/H^+^ exchanger and basolateral Na^+^/K^+^‐ATPase activity in the tubular cell membranes by bile acids 3, 16. Improvement of renal function may take several weeks determined by the severity of proximal tubular injury and bile cast formation 3, 11. The tubulopathic process is essentially correctable if the increase in bilirubin blood levels is curtailed in the early course of disease 4, 16, 17. This renal function improvement is delayed, nevertheless, if there is substantial intratubular bile cast formation.

Bile cast presence has been found to be more prevalent in the distal nephrons but is developed in more proximal tubules with the greater the severity of hyperbilirubinemia 18. The extent of intratubular bile cast formation parallels with the severity of hepatic injury as assessed by serum alkaline phosphatase and total bilirubin. Likewise, the number of bile casts encountered during kidney biopsies has been found to correlate with the severity of acute tubular injury 4, 19. In jaundiced patients, macroscopic findings include yellowish discoloration (bile staining) of the kidneys, which become dark green after formalin fixation.

Hemodynamic alterations causing prerenal azotemia may also be involved. High blood levels of bile salts have been proved to have negative chronotropic and inotropic effects, leading to cardiovascular compromise and reduced renal perfusion 20, 21. This is further aggravated by modifications in vascular reactivity supposed to be due to the widespread presence of endotoxemia, hypoalbuminemia, and nitric oxide‐mediated effects triggering systemic vasodilatation and renal vasoconstriction, giving rise to ischemic kidney injury 22.

Although the definite diagnosis of bile cast nephropathy can be accomplished by performing a kidney biopsy, such a procedure was contraindicated in our patient due to significant coagulopathy. Fortunately, careful bedside evaluation by urinalysis revealed numerous bile casts and muddy brown granular casts, which are pathognomonic for bile cast nephropathy and acute tubular necrosis (ATN), respectively. Muddy brown granular casts may be absent in 20–30% of patients with ATN. Moreover, FE_Na_ higher than 2%, FE_urea_ higher than 35%, and the presence of isosthenuria are also suggestive of acute tubular injury. Traditionally, ATN can be classified into two distinct types: ischemic ATN and nephrotoxic ATN. Nephrotoxic ATN is caused by direct tubular injury from endogenous or exogenous nephrotoxins. Myoglobin, hemoglobin, and light chains are notable endogenous substances associated with renal tubular toxicity 23. In addition, bile acids are potential endogenous nephrotoxins that cause renal tubular injury and bile cast nephropathy and should be regarded as a subtype of nephrotoxic ATN. The utility of performing a kidney biopsy in patients with established risk factors for bile cast nephropathy and ATN is unknown, and this procedure is rarely performed in clinical practice. It has been suggested that urine analysis is more practical and useful for diagnosis than invasive investigations in patients with jaundice‐related renal dysfunction 24.

## Conclusion

In conclusion, bile cast nephropathy should be considered in patients with acute kidney injury, severe hyperbilirubinemia, and/or evidences of proximal tubular dysfunction. The main pathophysiological mechanism of renal injury in bile cast nephropathy is direct nephrotoxicity of bilirubin and bile acids to the renal tubular epithelium and mechanical intratubular cast obstruction. Treatment of bile cast nephropathy consists mainly of supportive care and therapies targeting the underlying disease. Some case reports show that plasma absorption perfusion consisting of a bilirubin‐specific adsorbent resin may be effective 25, 26. Although the diagnosis of bile cast nephropathy can be confirmed by a kidney biopsy, the diagnosis can also be strongly suggested by examining urine samples for characteristic bile‐stained granular casts, which is a simple and inexpensive method.

## Conflict of Interest

The authors report no relevant conflict of interests.

## Authorship

KK: contributed to treatment planning, and the preparation, review, and submission of the manuscript. ST: contributed to the preparation of quality image files, and reviewed the manuscript. PW: contributed to the preparation of patient profiles, and reviewed the manuscript.
